# Learning by Observation: Insights from Williams Syndrome

**DOI:** 10.1371/journal.pone.0053782

**Published:** 2013-01-10

**Authors:** Francesca Foti, Deny Menghini, Laura Mandolesi, Francesca Federico, Stefano Vicari, Laura Petrosini

**Affiliations:** 1 Department of Developmental and Social Psychology, University “Sapienza” of Rome, Rome, Italy; 2 IRCCS Santa Lucia Foundation, Rome, Rome, Italy; 3 Child Neuropsychiatry Unit, Neuroscience Department, “Children’s Hospital Bambino Gesu’”, Rome, Rome, Italy; 4 School of Movement Sciences (DiSIST), University of Naples “Parthenope”, Naples, Italy; 5 Department of Psychology, University “Sapienza” of Rome, Rome, Italy; Vanderbilt University, United States of America

## Abstract

Observing another person performing a complex action accelerates the observer’s acquisition of the same action and limits the time-consuming process of learning by trial and error. Observational learning makes an interesting and potentially important topic in the developmental domain, especially when disorders are considered. The implications of studies aimed at clarifying whether and how this form of learning is spared by pathology are manifold. We focused on a specific population with learning and intellectual disabilities, the individuals with Williams syndrome. The performance of twenty-eight individuals with Williams syndrome was compared with that of mental age- and gender-matched thirty-two typically developing children on tasks of learning of a visuo-motor sequence by observation or by trial and error. Regardless of the learning modality, acquiring the correct sequence involved three main phases: a detection phase, in which participants discovered the correct sequence and learned how to perform the task; an exercise phase, in which they reproduced the sequence until performance was error-free; an automatization phase, in which by repeating the error-free sequence they became accurate and speedy. Participants with Williams syndrome beneficiated of observational training (in which they observed an actor detecting the visuo-motor sequence) in the detection phase, while they performed worse than typically developing children in the exercise and automatization phases. Thus, by exploiting competencies learned by observation, individuals with Williams syndrome detected the visuo-motor sequence, putting into action the appropriate procedural strategies. Conversely, their impaired performances in the exercise phases appeared linked to impaired spatial working memory, while their deficits in automatization phases to deficits in processes increasing efficiency and speed of the response. Overall, observational experience was advantageous for acquiring competencies, since it primed subjects’ interest in the actions to be performed and functioned as a catalyst for executed action.

## Introduction

In humans and other animals new competencies may be learned through active experience and through observation of others’ experience [1], [2]. Observing another person performing a complex action accelerates the observer’s acquisition of the same action and limits the time-consuming process of learning by trial and error [3]–[5]. Indeed, observational learning does not just involve copying an action and requires that the observer transforms the observation into an action as similar as possible to the model in terms of the goal to be reached and the motor strategies to be applied [5]–[10].

Observational learning is already present at birth [5], [11]–[13] and it is crucial for developing complex abilities such as language, social responsiveness, use of instruments to get things done [9], [14]. Thus, in children, learning new competencies by observing adults or peers is a central process in cognitive development [15].

**Figure 1:**
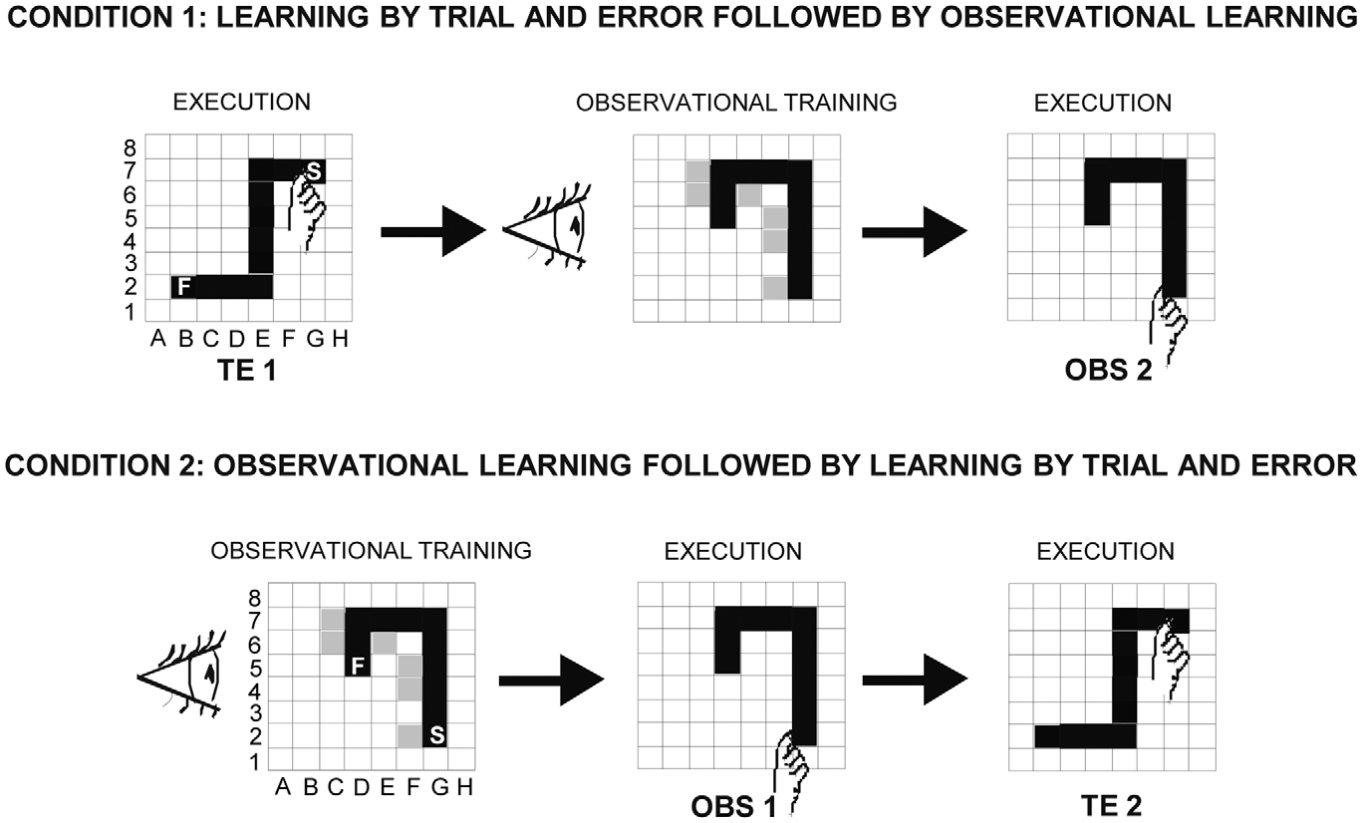
Schematic diagrams of the two experimental conditions. Condition 1: Learning by Trial and Error followed by Observational Learning: participants detected a sequence by trial and error (TE1), then they observed an actor detecting a sequence different from the one they had previously detected (observational training) and, finally, they reproduced the observed sequence (OBS2). Condition 2: Observational Learning followed by Learning by Trial and Error: participants were submitted to an observational training, then they reproduced the observed sequence (OBS1) and, finally, detected by trial and error a different sequence they had never observed (TE2). The incorrect positions touched by the actor during the observational training are evidenced in grey. S: starting point; F: final point.

By using an innovative task based on learning to detect a visuo-motor sequence, we demonstrated that in the presence of dyslexia the ability to learn by observation a previously observed visuo-motor sequence was markedly impaired, while the ability to detect a correct sequence by trial and error was preserved [16]. In the present research we focused on a population with learning as well as intellectual disability (ID), the Williams syndrome (WS) whose well-known neuropsychological profile with specific points of strengths and weaknesses allowed singling out cognitive processes working as learning went by. WS individuals show severely impaired visuo-spatial processing, planning and implicit learning [17]–[22], while they exhibit relatively preserved perception of the visual characteristics of objects and face recognition [23]. WS individuals have specific difficulty in maintaining visuo-spatial information in working memory and in performing long-term memory tasks [24], [25], consistently with a deficit of dorsal stream. Considering that the visuo-motor task to be learned by observation required to translate visual information into action, specific function of dorsal stream network [26], [27], WS individuals appear to be the ideal participants to investigate the cognitive processes involved in the observational learning. Performances of a group of WS individuals were compared with those of a mental age- and gender-matched group of typically developing (TD) children on a task requiring the learning of a visuo-motor sequence. The participants learned the sequence either by performing the task after observing an actor detect the sequence of correct items by trial and error (observational training) or by actually performing the task by trial and error (Fig. 1).

## Materials and Methods

### Participants

Twenty-eight WS participants and 32 TD children (used as controls) matching the WS individuals for mental age (MA) have been examined in the present study constituted by two experimental conditions: Learning by Trial and Error followed by Observational Learning (Condition 1); Observational Learning followed by Learning by Trial and Error (Condition 2) (Table 1). Only WS individuals with mental age (MA) of at least 5 years were included in the present research because participants with inferior MA did not succeed in completing the task. No significant differences in chronological age (CA), MA and IQ (*P* always >0.2) among participants performing Conditions 1 and 2 were found (Table 2).

**Table 1:** Description of WS groups (WS1 and WS2) and TD groups (TD1 and TD2) performing the two different experimental conditions.

Condition 1: Learning by Trial and Error followed by Observational Learning					
Group	Number	Gender	CAMean ± SEM	MAMean ± SEM	IQMean ± SEM
WS1	14	9 M	19.83±1.42	6.52±0.16	54.87±1.69
TD1	16	11 M	6.78±0.15	7.02±0.28	106.12±2.51
**Condition 2: Learning by Observation followed by Learning by Trial and Error**					
WS2	14	8 M	17.64±1.37	6.60±0.19	53.68±1.36
TD2	16	8 M	6.76±0.11	7.40±0.28	111.62±2.06

CA: Chronological Age.

MA: Mental Age.

IQ: Intelligence Quotient.

**Table 2:** Comparisons of chronological age (CA), mental age (MA) and Intelligence Quotient (IQ) between WS groups (WS1 and WS2) and TD groups (TD1 and TD2) that performed the two different experimental conditions.

Group	CAMean ± SEM	F (freedom degrees)	MAMean ± SEM	F (freedom degrees)	IQMean ± SEM	F (freedom degrees)
WS1	19.83±1.42	(1,26)0.61*P = 0.43*	6.52±0.16	(1,26)0.04*P = 0.83*	54.87±1.69	(1,26)0.15*P = 0.70*
WS2	17.64±1.37		6.60±0.19		53.68±1.36	
TD1	6.78±0.15	(1,30)0.005*P = 0.94*	7.02±0.28	(1,30)0.45*P = 0.50*	106.12±2.51	(1,30)1.43*P = 0.23*
TD2	6.76±0.11		7.40±0.28		111.62±2.06	

The clinical diagnosis of WS was confirmed by fluorescence *in situ* hybridization (FISH) genetic investigation, which showed the characteristic deletion on chromosome band 7q11.23. WS participants were part of a larger pool of individuals with learning disabilities attending the Children’s Hospital Bambino Gesù of Rome for clinical and rehabilitative follow-up. All of them lived at home with their families. The parents of all individuals who participated in the study provided written informed consent. This study was approved by the Ethic Committee of the Children’s Hospital Bambino Gesù of Rome and conducted according to the Helsinki declaration.

WS individuals were tested in a quiet room at the Children’s Hospital Bambino Gesù. TD children were individually tested in a quiet room at their schools.

### Intelligence Evaluation and Neuropsychological Assessment

In the present study, the brief version of the Leiter International Performance Scale–Revised [28] was employed (four out of 10 subtests: Figure Ground, Form Completion, Sequential Order and Repeated Patterns). The brief IQ and the corresponding mental ages were computed. Visuo-motor integration [29], visuo-spatial perception [30] and memory [31] were assessed (Table 3).

**Table 3:** Statistical comparison of visuo-spatial performances of WS and TD participants.

	WSMean ± SEM	TDMean ± SEM	Effect	F_(1, 58)_ value	*P*
Visuo-motor integration	13.39±0.50	15.25±0.26	Group	11.37	0.0013
Visuo-spatial short-term memory (VSS)	2.79±0.20	3.53±0.14	GroupTaskInteraction	8.299.634.82	0.00550.00290.032Newman–Keuls testVSS: 0.00021VOS: 0.36
Visuo-object short-term memory (VOS)	2.64±0.12	2.91±0.11			
Visuo-perception test – Spatial (VPT-S)	15.18±1.07	18.03±.77	GroupTaskInteraction	7.0665.754.06	0.010<0.00010.048Newman–Keuls testVPT-S: 0.00038VPT-F: 0.31
Visuo-perception test – Form (VPT-F)	11.32±0.36	12.16±0.26			

### Experimental Procedure

Each participant was sat in front of a computer touch screen (distance 60 cm). In both Conditions, the experimenter acting as the actor (F.F.) was sat near the participant. A 8×8 black matrix appeared on the touch screen. The subject was asked to find a hidden sequence of “correct” squares prepared in advance by the experimenters. The sequence was composed of 10 adjacent spatial positions in the matrix, which formed a “snake-like” pattern (Fig.1). To explain the task to each participant the experimenter used the same verbal instructions: “You have to find a route formed by ten squares. When you touch a correct square it will be turned grey and you will hear a sound; conversely, if you touch a wrong square, it will be turned red. In this case, you have to find a new grey square. You have to start the route each time you find a new correct square. After finding the whole route, you have to re-touch it three times without making lighted red squares”. The participants started touching a grey square, which was the first element of the sequence and was always lit up. In the search for the second correct square, the participants had to touch one of the four squares bordering the grey square by moving in the matrix vertically or horizontally, but never diagonally. Each touched square (correct or incorrect) was lit up for 500 ms and then lighted off again; thus, no trace of the performed sequence remained on the screen. In learning the sequence by trial and error, the participants tried to find the correct sequence immediately after the verbal instructions. Conversely, in the observational learning task after the verbal instructions the participants observed the experimenter while she detected a 10-item sequence by trial and error (observational training). The experimenter performed the task by always making the same errors in the same positions, so that all participants observed the same pattern of correct and incorrect touches. Two minutes after the end of the observational training the participants were required to actually reproduce the observed correct sequence.

The tasks involved three phases: the Detection Phase (DP) that ended once the participants found the tenth correct position; the Exercise Phase (EP) in which they had to repeat the 10-item sequence until their performance was error-free; the Automatization Phase (AP) that ended when the correct sequence was repeated three consecutive times without errors.

### Parameters

Error parameters: *DP errors,* calculated as the number of incorrect items touched in detecting the ten correct positions; *EP repetitions,* calculated as the number of replications needed to reach the error-free performance. Time parameters: *AP times* (in msec), calculated as the time spent carrying out each of the three repetitions of the sequence.

### Analysis of Error

To assess the kind of error further parameters were taken into account considering the two phases DP and EP together: the number of *sequence errors*, as touching a “correct” square in “wrong” moment (e.g. touching E7 before than F7); *side-by-side errors*, as touching the squares bordering the “correct” sequence (e.g. E8); *illogical errors*, as touching any other square (e.g. B5); *perseverations,* as consecutively touching the same item or a fixed sequence of items. Furthermore, in the task of observational learning we calculated the number of *imitative errors*, as touching the same squares wrongly touched by the actor during the observational training (e.g. F4) (Fig.1).

### Condition 1: Learning by Trial and Error Followed by Observational Learning

Fourteen WS and 16 TD individuals (Table 1) firstly detected a sequence by Trial and Error (TE1) and, after ten minutes from task end, they were submitted to the observational training. After two minutes, participants were required to actually reproduce the observed sequence (OBS2). There was no fixed time limit for executing the task.

A pilot study was conducted to verify if the two sequences arranged to be used as “TE” and “OBS” sequences did not differ as to degree of difficulty. Six TD children [3 M] of mental age 6.10±0.3 detected the two different sequences by trial and error; presentation order was randomized among participants. Errors made in detecting each sequence were calculated by one-way ANOVA with repeated measures. The analysis failed to reveal any significant difference between sequences (*F*_(1,5)_ = 0.63, *P* = 0.46), confirming sequences of the same difficulty.

### Condition 2: Learning by Observation Followed by Learning by Trial and Error

Fourteen WS and 16 TD individuals (Table 1) first observed the experimenter detect a sequence (OBS1) and then actually reproduce it. After ten minutes from task end, they detected a different sequence by trial and error (TE2). Thus, the difference of the two conditions was that participants reproduced a sequence learned by observation *after* (Condition 1) or *before* (Condition 2) the detection of a different sequence by trial and error.

To evaluate mental representative mapping abilities, at the end of the reproduction of the sequence participants were required to draw the arrangement of the sequence on a 8×8 matrix sketched on a paper sheet. Thus, any participant drew the arrangement of two sequences, one learned by observation and the other one by trial and error. Mapping abilities were evaluated by tabulating the variable “errors” into three categories: “no error”, “one error” and “more than one error”.

### Attentional Task

The sustained attentional abilities of all participants were tested. Participants sat in front of a computer monitor and were required to put their left index fingers on the A key of the keyboard and to put their right index fingers on the L key. The visual stimulus was a grey circle presented on monitor center for a duration varying from 1400 (short) to 2600 (long) msec in steps of 200 msec in a randomized order. Participants were submitted to a brief training in which they were instructed to judge 20 stimuli as short or long and to press the A or L keys, respectively. In the testing phase the participants had to judge the duration of 70 stimuli (10 stimuli of each of the 7 durations) and to press the A or L keys as quickly as possible after the stimulus appeared. The computer program recorded reaction times (with 1-ms resolution) and accuracy of the response. The responses were then analyzed by clustering them in blocks of ten (regardless of stimulus duration) (i.e. 1–10, 11–20, 21–30….61–70).

### Statistical Analyses

The data were first tested for normality (Shapiro-Wilk normality test) and homoscedasticity (Levene test) and then compared by using two-way, three-way or four-way analyses of variance (ANOVAs). The two-way ANOVAs were performed by applying the mixed model for independent variable (group) and repeated measures (error, square or block). Three-way ANOVAs (group×condition×task) were performed on most parameters, while the four-way ANOVA on the three AP times was performed by applying the mixed model for independent variables (group, condition and task) and repeated measures (time). These analyses were followed by post-hoc multiple comparisons using Newman–Keuls test. In evaluating mapping abilities the error categories were analyzed by Chi-Square.

Because the 28 WS participants were differently aged (N = 9 age range: 8;9–14;1; N = 10 age range: 14;9–19;9; N = 9 age range: 22;9–35;3), we verified the sample homogeneity by comparing the performances of three differently aged WS sub-groups on three main parameters of the learning tasks they performed (DP errors; EP repetitions and AP times) by using MANOVAs. These analyses revealed no significant difference among WS sub-groups’ performances. Namely, in the tasks of learning by trial and error (TE1–TE2), the MANOVA revealed a not significant sub-group effect (F_(2,25)_ = 0.12, *P* = 0.88) and a significant parameter effect (F_(2,50)_ = 154.54, *P*<0.0001). The interaction was not significant (F_(4,50)_ = 0.13, *P* = 0.96). In the tasks of observational learning (OBS1–OBS2) the MANOVA also revealed a not significant sub-group effect (F_(2,25)_ = 0.47, *P* = 0.62) and a significant parameter effect (F_(2,50) = _85.46, *P*<0.0001). The interaction was not significant (F_(4,50)_ = 0.47, *P* = 0.75). Thus, we pooled together the 28 differently aged WS individuals.

All statistical analyses were performed by using Statistica 8.0 for Windows and the significance level was established at *P<*0.05.

## Results

### Learning Tasks

**Figure 2:**
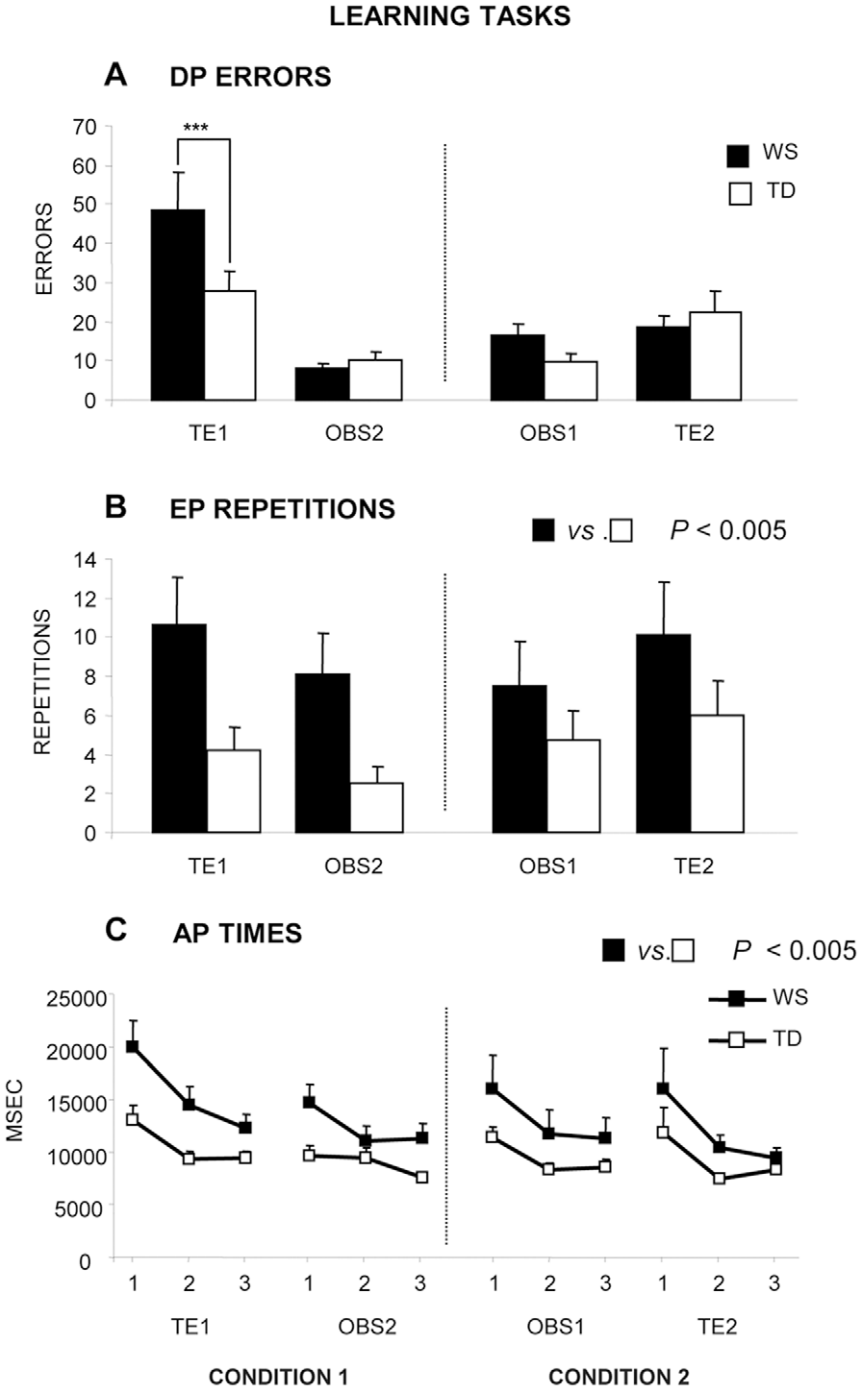
Performances exhibited by WS and TD participants in the two experimental conditions. DP: Detection Phase; EP: Exercise Phase; AP: Automatization Phase. Data are expressed as mean ± SEM. The asterisks indicate the significance level of post hoc comparisons between groups (***P<0.001).

WS participants performed a number of DP errors not significantly different from TD children after the observational trainings (OBS1–OBS2) and were significantly impaired in detecting the sequence by trial and error in TE1 compared with any other intra- or inter-group condition (Fig. 2A), as revealed by post-hoc comparisons (always *P*<0.001) on the second-order interaction (F_(1,56)_ = 8.37, *P = *0.0054) of the three-way ANOVA (group×condition×task).

In EP, when individuals repeated the sequence until their performance was error-free, WS participants needed a significantly higher number of repetitions in comparison to TD children regardless of condition (1 or 2) and trial (OBS or TE), as revealed by the group effect (F_(1,56)_ = 9.58, *P* = 0.0030) of the three-way ANOVA (Fig. 2B). The analysis of the three AP times revealed that although all participants exhibited significantly reduced times as the task went by (F_(2,112)_ = 27.62, *P*<0.00001), WS individuals were significantly slower than TD children (F_(1,56)_ = 10.37, *P* = 0.0021), revealing a difficulty in automatizing the sequence (Fig. 2C).

### Analysis of Error

**Figure 3:**
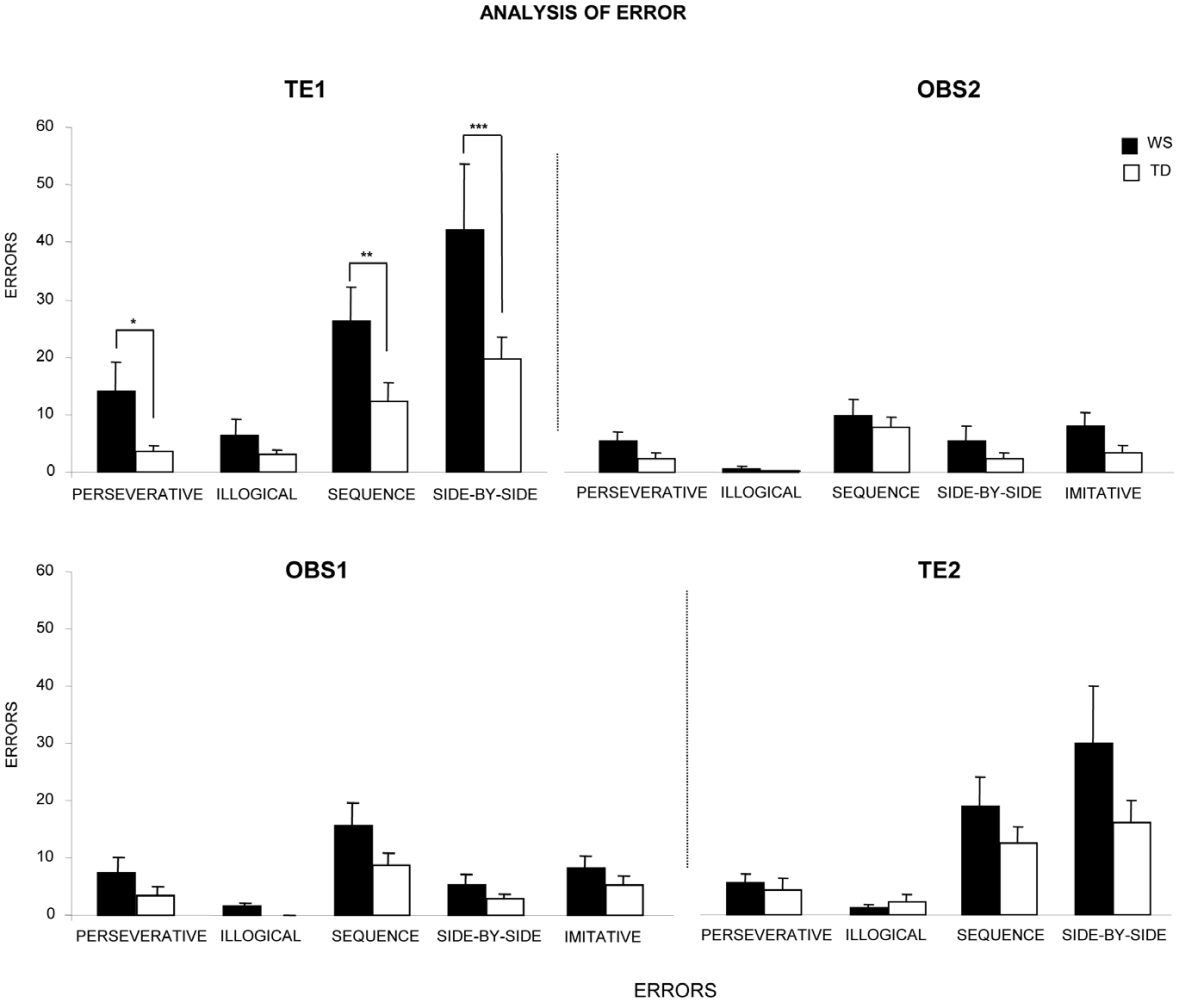
Errors exhibited by WS and TD participants in the two experimental conditions. Data are expressed as mean ± SEM. The asterisks indicate the significance level of post hoc comparisons between groups (*P<0.05; ** *P*<0.005; *** *P*<0.001).

**Figure 4:**
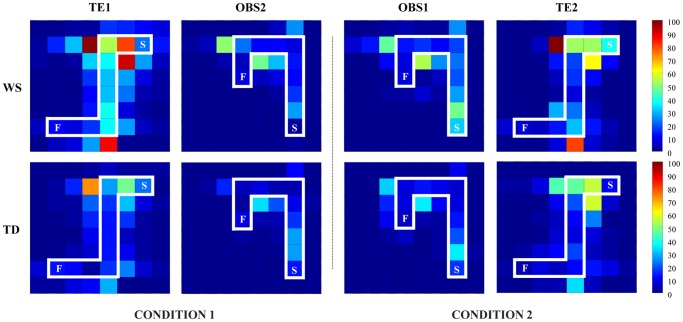
Incorrect items touched on the screen by WS and TD participants in performing the tasks. On the right, the chromatic scale indicates the sum of incorrectly touched items (brown and blue denote maximal and minimal values, respectively). S: starting point; F: final point.

In TE1, although WS and TD participants did not differ in the number of illogical errors, WS individuals exhibited values of sequence, side-by-side and perseverative errors higher than TD children, as revealed by post-hoc comparisons made on the interaction (F_(3,84)_ = 3.14, *P* = 0.029) of the two-way ANOVA (group×kind of error) (Fig. 3). The highest number of sequence errors of WS individuals was found in E7 and F7 squares when a change of strategy was required (i.e. after an error re-starting the sequence from the first item rather than continuing along on the “snake”) (Fig. 1), as revealed by post-hoc comparisons made on the interaction (F_(9,252)_ = 1.96, *P* = 0.044) of the two-way ANOVA (group×square) (Fig. 4). As for side-by-side errors, the high number of errors of WS individuals was due to their significantly more frequent touching of a wrong square when a change of direction was required (squares: D7, F6, E1) (Fig. 1), as revealed by post-hoc comparisons made on the interaction (F_(27,756)_ = 2.42, *P*<0.0001) of the two-way ANOVA (group×square) (Fig. 4).

The analysis of error in the remaining tasks (OBS2, OBS1 and TE2) revealed no significant difference between groups, even if significant difference among kind of errors was found (always *P*<0.00001) (Fig. 3).

### Mapping Abilities

Mental representative mapping abilities of the participants were evaluated by having them draw the arrangement of sequences they had just performed. No significant difference among categories of errors and between groups was found in any sequence (*P* at least >0.4).

### Attentional Task

**Figure 5:**
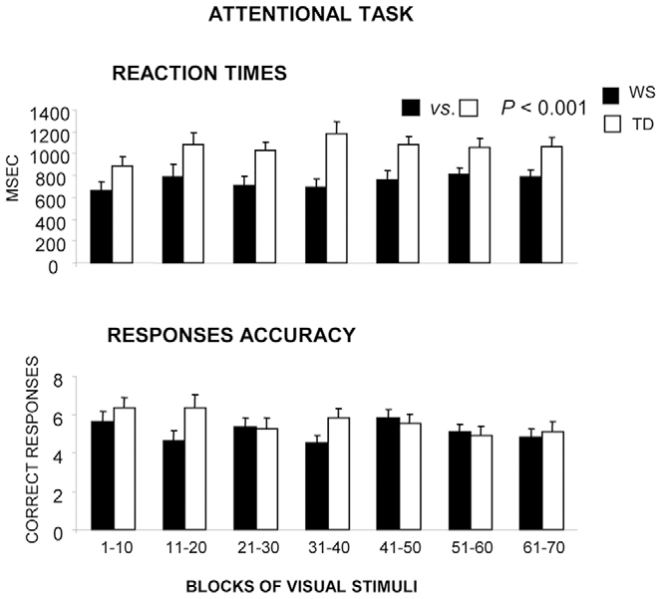
Performances exhibited by WS and TD participants in the Attentional Task. Data are expressed as mean ± SEM.

Two-way ANOVAs (group×block) on reaction times or response accuracy of the WS and TD groups revealed no attentional decay in both groups, as indicated by not significant difference in the reaction times in the seven blocks (F_(6,348)_ = 1.55, *P* = 0.15). A similar result was obtained when response accuracy was analyzed (F_(6,348)_ = 1.80, *P* = 0.10). Notably, a significant difference was found between WS and TD groups on reaction times (F_(1,58)_ = 13.52, *P* = 0.00051), given WS participants pressed the keys at the appearance of the stimulus more quickly than TD children (Fig. 5).

## Discussion

Our study adopted a matched-group design to determine whether the learning performance of WS individuals was above or below that expected given their general level of intellectual functioning indexed as MA. However, although this design is one the most commonly employed measures of matching in ID research, we are aware that it has limitations in respect to ID-matched control group design that takes into account the cognitive profile of the specific pathology. Nevertheless, even the ID-matched control group cannot be taken as a guarantee of normative group, due cognitive profiles among different etiological groups with ID exhibit different peaks and troughs [32]. In an attempt to overcome the difficulties in matching individuals of different groups on any one particular measure it has been proposed the use of regression techniques that take the factors related to task performance into account [32]. However, this measure requires specific statistical properties of the data (as homogeneity of regression slopes or sample size), hardly available in studies on population affected by rare genetic conditions as WS.

The present study documented as WS participants significantly beneficiated of observational training as TD MA-matched children. This was true specifically in the DPs of learning tasks, while as for EPs and APs, in all tasks regardless of presentation order (1 or 2) or learning modalities (OBS or TE), WS participants performed significantly worse than TD children. The powerfully positive effect of observational training was present not only in reproducing the previously observed sequences (OBS1 and OBS2) but also affected the subsequent detection of a sequence by trial and error (TE2). However, the practice effect, inevitably present in any second task, potentially could affect performances.

Since WS individuals exhibit difficulties in maintaining visuo-spatial information in working memory and in performing spatial long-term memory tasks (Table 3) [20], [24], [25], their heavily impaired performances in all EPs appear linked to spatial working memory deficits and difficulties in bringing together the short sequences detected during DP, in maintaining them in working memory to recall the whole sequence trace and in monitoring the correct execution of the sequence. These findings indicate that the observational training exerts beneficial effects mainly on the acquisition of strategies to be applied.

In both Conditions, WS participants displayed AP times longer than TD children, even if progressively diminishing as the task went by. This finding was not a consequence of the fine motor deficits usually reported in WS individuals. In fact, consistently with Vicari et al. (2007) [22], the reaction times exhibited by WS group in the Attentional Task were even shorter than those of TD group. Thus, the longer WS times were related to deficits in automatization processes increasing efficiency and speed of the response to reach highest levels of performance [33]. Automatizing skills is mainly linked to the functions of sub-cortical structures, as the cerebellum and basal ganglia and to their bidirectional interconnections with cortical structures [34], [35], [36], [37]. The cause of automatization and procedural deficits of WS individuals could be their remarkable hypoplasia of the basal ganglia [38] and the disproportionate enlargement of the cerebellum [39], [40], [41], [42], [43]. Indeed, in WS individuals skill-learning abilities are impaired, as revealed by their performance in Tower of London test [21], Serial Reaction Time task [22] and Radial or Multiple Reward Mazes [44], [45], [46].

By analyzing the kind of errors, some remarks can be made. First of all, both groups made a very low number of illogical errors, thus suggesting all participants similarly managed task fundamentals (Fig. 3 and 4). As for imitative errors, no difference between groups was found, thus suggesting participants did imitate but did not hyperimitate [47]. Conversely, WS individuals made more sequence and side-by-side errors than TD children in TE1, particularly when a change of direction was required. Errors in stopping the more easy “keep-straight” response and performing the more demanding “turn-left” response resulted by the WS difficulty in suppressing a previously correct but then inappropriate response. Correctly responding requires executive control processes based on frontal lobe function, as response inhibition, cognitive flexibility and attentional shifting [48], [49], [50], [51]. WS individuals are impaired in spatial planning, working memory, cognitive flexibility and inhibiting well-learned responses become inappropriate to the situation [52]. Indeed, the executive function deficits that impaired WS performance dramatically reduced after the observational training, once more indicating the teaching power of the observation. WS participants made perseverative errors that could result from difficulties in withholding the inappropriate repetition of a response despite knowing that it was not the correct one. This is an important component of top-down executive control. Notably, perseverations may be symptom not only of prefrontal dysfunction but also of cerebellar and basal ganglia damage provoking “frontal-like” cognitive deficits [34], [53], [54], [55], [56], [57].

The prevalently frontal processes require a modulation in more posterior brain systems, via the attention networks. Basic aspects of attentional processing are selective spatial attention that allows maintaining the focus of processing between spatial locations, and the attentional processing that allows a kind of “selection for action” [58], [59], [60]. Namely, the action of reaching the right square required attentional modulation to plan, select and initiate the appropriate behavior, to direct it toward the selected goal, and to inhibit actions inappropriate for the current goal. Because many brain structures that are part of the attention networks are included within the dorsal stream network [27], it is not surprising that WS participants performed more errors when behavioral inhibition and attentive shifting were required but no help from observing the actor was provided [61]. The “dorsal-stream vulnerability” in WS [62] is manifested not only in the spatial and visual processing occurring within the occipital and parietal areas but also in the processing of spatial information by frontal control systems, as reported in an fMRI study [63].

At the end of testing, mental representative mapping abilities were evaluated by drawing the arrangement of squares just discovered. WS and TD participants were similarly able to represent the shape of the “snake”. This finding is consistent with the observation that WS individuals exhibit no difficulty in mentally visualizing objects without spatially manipulating them [25], [64] and supports the indication that the present learning protocols encompassed requests of visual imagery.

If observative experience functions as a catalyst for executed action, it can be advanced that observing a sequence prior to experiencing it primes subjects’ interest in the actions to be performed to detect rules and sequence. In fact, the present results indicate that the observation of action has a strong impact on action memory. The influence of action perception on action production requires cross-modal information be coordinated. Action and perception share the distal reference and are coded in a common representational medium [65], so that perceiving an action activates the corresponding motor representation within the observer automatically and without conscious effort [66], [67], [68].

The close interplay between observation and execution of actions found in the present study is supported by studies providing evidence of a striking overlap in the brain systems recruited for one’s own action, observation of others’ action and imitation of action [69]. In particular, when imitation is aimed at learning novel actions, the activation of the “core circuit for imitation” [70] involving the inferior frontal gyrus, the inferior parietal lobule and the superior temporal sulcus seems to be integrated with activation of the dorso-lateral and ventro-medial prefrontal cortex, for selection of motor acts and error prediction [71], of the premotor areas, for motor preparation [70], [72], [73] as well as of cerebellar areas, whether or not it is accompanied by actual motor acts [74], [75], [76].

The existence of direct feed-forward connections from perceptual to motor processes allows observation sculpting motor abilities by exploiting the functional overlapping between perception and action systems. It has been suggested that observation of actions engages motor-related processes similar to those of actual execution, promoting the development of an efference copy of the descending motor commands, which in combination with a forward model provides a prediction of sensory consequences [77], [78], [79], [80]. Thus, action observation, efficiently translated into the matching motor representation, powerfully activates the feed-forward predictive processes, so that learning does occur. Notably, even in WS individuals the beneficial effect of observation was evident although linked only to the DP. Action observation seems to result in an amelioration of frontal functions, as motor strategy planning, decision-making processes or response inhibition needed to guide planned sequential actions. Thus, observational training allows the acquisition of the strategies to be applied to identify and learn the visuo-spatial sequence. Notably, when the observation did not play any role (as in the DP of TE1), frontal deficits markedly affected WS performance. However, it has to be underlined that as far as the observational training was beneficial, in WS individuals it did not succeed in smoothing out the deficits in processing visuo-spatial information mainly linked to their repeatedly described dorsal stream vulnerability [62].
